# Facts and fears that limit digital transformation in farming: Exploring barriers to the outreach of wireless sensor networks in Southwest Iran

**DOI:** 10.1371/journal.pone.0279009

**Published:** 2022-12-16

**Authors:** Fatemeh Taheri, Marijke D’Haese, Dieter Fiems, Hossein Azadi

**Affiliations:** 1 Department of Agricultural Economics, Ghent University, Ghent, Belgium; 2 Department of Telecommunications and Information Processing, Ghent University, Ghent, Belgium; 3 Department of Economics and Rural Development, Gembloux Agro-Bio Tech, University of Liège, Gembloux, Belgium; 4 Faculty of Environmental Sciences, Czech University of Life Sciences Prague, Prague, Czech Republic; 5 Faculty of Environmental Science and Engineering, Babeș-Bolyai University, Cluj-Napoca, Romania; Canakkale Onsekiz Mart University, TURKEY

## Abstract

Wireless sensor networks (WSNs) are considered part of an environmentally friendly technology leading to more timely and cost-effective production and management of farms. Despite the potential of WSNs to agricultural development in the global South, outreach is still very limited, also in Iran. Therefore, in order to facilitate the adoption of WSNs, it is necessary to identify the factors influencing and challenging the adoption of this technology. This exploratory study uses a qualitative approach to identify the main barriers WSN outreach is facing. In the results, we distinguish facts that we define as issues or barriers that were identified by others from fears that are not supported by evidence so far, at the level of the farmers, the government actors as well as the technology itself. Facts include communication barriers such as internet access, farmers’ knowledge levels and rigidity to change as well as the government’s top-down organisation of the extension programme and support levels. Fears are mainly expressed on the technology itself and relate to costs, a lack of access, the complexity of use and reliability of the data. We provide a nuanced view of how fears need to be acknowledged and facts are to be tackled.

## 1 Introduction

All over the world, the agricultural sector aims at producing more food from less land by using natural resources more efficiently to reduce the impact on the environment and simultaneously, to meet the increasing demand for food in the coming decades [1]. In response to concerns about food security and the degradation of natural resources in agriculture, various policies and agricultural strategies are developed and promoted [2]. Among others, precision agriculture (PA) is increasingly put forward as a sustainable farming method for efficient resource utilisation and increased food production stability [3]. One of the key tools in the PA technological package are the wireless sensor networks (WSNs) that can offer farmers processed real-time field data from sensors dispersed over their plots [4]. WSNs allow for a more precise application of resources [5] and as such can play an important role in managing water resources for irrigation, understanding crop changes to evaluate the optimal harvesting point, estimating pesticide and fertiliser requirements and predicting crop performance more accurately [6]. Moreover, WSNs are generally a relatively low-cost investment, which increases the probability of their adoption on farms [7]. As a result, the application of WSNs is showing fast progress in the agricultural sector [8, 9].

Studies show that WSN applications are equally promising for agriculture in the Global South [10–13]. Dube [14] investigated how Ethiopian farmers could monitor their farms using their mobile phones in conjunction with WSNs. He found that a technology based on mobile communication and WSNs creates farm field monitoring and plant protection systems resulting in sustainable crop production and poverty reduction. El-kader and El-Basioni [15] discussed how using WSNs in Egyptian potato farming could improve production and storage by managing the risks of disease incidence and the presence of harmful fungi. Fourati et al. [16] proposed a web-based decision support system communicating with a WSN to monitor solar radiation, humidity, rain and temperature that provides input to schedule irrigation of the olive fields in Tunisia. Mafuta et al. [10] showed the application of WSNs in the rural areas of Malawi. Ali [12] has set up a real-time WSN monitoring system in Africa that can send information about soil moisture levels obtained by sensors to the farmers’ mobiles. Despite the great potentials of WSNs, this technology is often disregarded and considered a perhaps overly complex technological intervention for developing countries [13].

Although the World Bank [17] assigns Iran’s economy into the upper-middle-income group, its agriculture sector is still underdeveloped [18] and challenged by water stress, nutrient deficiencies, weeds, insects and crop diseases that lead to lower agricultural productivity, production stability and farmer income [19]. To overcome these challenges and improve farm productivity, a new agricultural strategy is required [20]. Iran’s Sixth Five-Year Development Plan (2016–21) thereto inscribes the promotion and extension of digital and improved technologies. This may include WSNs, which, despite their potential, are barely adopted in Iran. There is clear evidence that where WSNs are used, water, seed, and fertilizer use can go down significantly. This would eventually lead to less demand for inputs and land. So, the technology is becoming increasingly important to small-scale farmers, particularly in resource-poor and risk-prone farming settings. However, due to cost and technical constraints, its adoption in these areas has been limited. Morever, these constraints (and hence barriers) are not sufficiently understood to allow for an effective strategy to promote WSN use amongst the farmers. Barriers farmers face have been studied, yet for other technologies than sensors. For example, Chenani et al. [21] used a qualitative approach to identify farmers’ adaptation barriers to climate change. Their results explored and categorized numerous barriers to adaptation, which were social and cultural, economic, technological, informational, and market-based. Yazdanpanah et al. [22] identified cues to action, perceived barriers, general beliefs, and perceived benefits as the main factors affecting the farmers’ willingness to implement organic farming in southwest Iran. Rahimi-Feyzabad et al. [23] determined the institutional constraints to groundwater resource management.

This paper sets out to explore, identify and recognise the status and barriers to WSN adoption. We use a qualitative and open-ended approach to gather sufficient detailed information [24] to answer the following research questions (RQ):

RQ_1_: How would the respondents perceive the outreach of WSNs?
RQ_2_: How can WSNs be disseminated?
RQ_3_: What are the challenges of WSNs outreach in the agriculture sector?
RQ_4_: How would the respondents metabolize barriers to WSN outreach?

The remaining part of this paper is structured as follows: Section 2 provides an overview of the most related studies. Section 3 describes the methodology used in this study. Section 4 provides the results answering the four research questions of the study. It also discusses and compares the results with similar studies. And lastly, the study closes with Section 5, in which the whole work is concluded and the limitations of the study are summarized.

## 2 Literature review

A WSN system is a self-configuring network of small sensor nodes communicating among them using radio signals, and deployed in quantity to sense the physical world [25]. According to Chebbi et al. [26], there are four main components that make up a sensor node. The parts include a sensing unit, a processing unit, a transmission unit and a power unit. Depending on the type of application, a sensor node may have additional parts such as a position finding system, mobilize and a power generator. The sensing unit usually takes the burden of sensing and gathering sensor data and then passes the data to the processing unit. The processing unit receives the sensed data and processes it according to a set procedure or program. A transmission unit connects the sensor note with a network. The power unit supplies the power required to run a sensor node.

Cheap, smart sensors, networked through wireless links and deployed in large numbers, provide a wide range of opportunities for monitoring and controlling homes, cities and the environment [27]. WSN technology is expected to have a significant impact on our lives in the coming decade. In fact, billions of tiny devices with sensing, computation, and communicating capabilities are expected to be employed in a variety aspects of everyday life, including surveillance, environmental monitoring, smart grids and cities, connected cars, precision agriculture and healthcare [28]. Zemrane et al. [29] developed an Ehealth ecosystem based on a microcomputer connected to more than ten health sensors to measure, record and transmit health information of a patient to the database center in real time to understand the patient’s state of health and to warn the patient’s family and hospital consultant if necessary for prompt intervention. According to Darvishi et al. [30], sensor technologies empower Industry 4.0 by enabling the integration of in-field and real-time raw data into digital twins. The term Industry 4.0 refers to the fourth industrial revolution, which aims to boost automation through the effective combination of the Internet of Things, cyber-physical systems and cloud computing technologies. Within Industry 4.0, sensors play a crucial role by measuring various physical parameters, thus enabling monitoring, controlling and decision-support capabilities.

In recent years, many applications have been proposed for WSNs in agriculture. Table 1 provides an extract of studies on WSN applications in different aspects in agriculture. The use of WSNs technologies is belived to greatly improve agricultural resource management by providing access to information in real time in a super-connected way. Furthermore, WSNs technologies are expected to generate means that are extremely productive, and adaptable to changes such as those associated with climate change. As a result, it can help the Global South achieve higher food security, profitability and sustainability [31]. However, WSNs pose some challenges that need to be addressed for the long term viability of the created systems. Few studies consider the challenges of WSN adoption at the farm level, especially for small-scale farms [32–34]. In addition, these studies mostly focused on the technical aspect of WSNs and considered their technical limitation [35, 36].

**Table 1:** An extract of studies of studies conducted on WSN applications in agriculture.

Source /Authors	Location	Sensor technology	Communication technology	System boundary	Application	Specific Use	Work done
Adamsab et al., 2021 [40]	Oman	DS18B20, DHT sensors	WiFi	Arable farming	Monitoring soil & climate	Developing a smart irrigation system	Proposed an intelligent irrigation monitoring system
Assaf and Ishaq, 2020 [41]	Palestine	Soil moisture sensor, temperature sensor, DHT22 sensor	WiFi	Farmland	Monitoring soil	Automate the amount of water dispensed to the plants	Employed a microcontroller module to control the right amount of water that should be provided to the plant.
Chowdhury et al., 2020 [42]	Qatar	YF-S201 Hall-Effect flow sensor, Atlas Scientific pH Kit 0–14 pH sensor	WiFi	Indoor farming (hydroponics)	Monitoring soil & climate	Designing an automated indoor farming system	Designed an indoor automatic vertical hydroponic system.
Bamurigire et al., 2020 [43]	Rwanda	Water level sensors	GSM	Farmland	Monitoring irrigation water	Irrigation decision process	Used IoT sensors to automatically provide irrigation control according to seasonal and daily irrigational needs
Dahane et al., 2019 [44]	Algeria	NRF24L01	Radi module	Laboratory	Monitoring soil & climate	Automated irrigation system	Automated irrigation management platform using a wireless sensor network
Adetunji and Ngene, 2018 [45]	South Africa	TCS3200 color sensors	GSM/GPRS	Farmland	Monitoring crop	Measuring leaves’ droppings	Designed a monitoring system for leaves’ droppings for decomposition to manure.
Ahmed et al., 2018 [46]	Sudan	PH sensor, electrical conductivity sensor	XBee	Farmland	Monitoring soil	Irrigation and fertilization control	Designed an automated fertigation system based on the internet of things.
Burke and Lobell, 2017 [47]	Kenya	Terra Bella imagery	GPS	Farmland	Satellite imagery	Assessment of yield variation	Satellite-based assessment of maize yield variation
Duchemin et al., 2009 [48]	Morocco	FORMOSAT-2 images	GPS	None	Satellite imagery	Irrigation water amount estimation	Used machine learning models for prediction of irrigation water quality parameters
Bannari et al.,2007 [49]	Morocco	Advance Land Imaging (EO-1 ALI) sensor spectral bands EO-1 ALI	GPS	None	Monitoring soil	Saline and sodic soils characterization	Slight and moderate saline and sodic soils characterization using multispectral remote sensing

Some other studies talk about adoption. Hite and Hudson [37] conducted a telephone survey on 762 respondents in Mississippi, US, to assess the public willingness to pay for precision application equipment to reduce nutrient runoff. Their findings suggest public support exists for such policies. Aubert et al. [38] performed an empirical analysis of farmers’ adoption decisions of precision agriculture technology. The data was collected using a survey of 438 farm operators in Quebec, Canada. Their findings highlight the importance of compatibility among precision agricultural technology components and the crucial role of farmers’ expertise. Tohidyan Far and Rezaei-Moghaddam [20] investigated factors influencing agricultural consultants’ attitude and intention to use precision agricultural technologies. They employed data from a survey amongst 183 agricultural consultants to show that agricultural personnel and consultants intended to use precision agricultural technologies. Panaligan et al. [39] assessed the potential for adoption of WSN technology for irrigation water management in the Philippines. They conducted a survey with farm owners and farm managers of whom 83% expressed willingness to adopt the WSN technology if it is properly demonstrated and if it will give a high return on investment. These studies are mainly quantitative and deeper insights are missing which is what this paper address. In this paper, the interviews from experts and farmer groups are used to identify the facts and fears that explain why WSN adoption has been slower.

## 3 Methodology

### 3.1 Study design

Because the WSN technology is passing through a research stage in Iran, there were few farmers who knew or had experience with WSNs. Hence, reaching a large number of respondents who were able to participate in a survey proved extremely difficult. This study benefits from exploratory and qualitative methods. In contrast to quantitative studies, which often aim for a large sample size, a qualitative study typically uses data from a smaller sample. However, such low number of respondents should not bias our research because the main objective is in-depth understanding. This also means that we may not generalize the findings to all farmers in Iran. Yet, the findings are valid and useful for the province studied and possibly for other areas in the country with very similar characteristics in terms of climatic conditions, agricultural practices, and socio-cultural aspects. Most qualitative studies have around 5–50 respondents [50]. Abay et al. [51] conducted a qualitative study in which data were gathered through 11 in-depth interviews and five focus group discussions. In another qualitative study by Nguyen et al. [52], a total of 25 interviews were conducted to understand the farmers’ perception of climate change. In Cao et al. [53], ten qualitative, in-depth interviews were conducted. Liang et al. [54] in their study interviewed 12 participants, including farmers, cooperative professionals, and agricultural extension educators, to identify the potential impact of agricultural cooperatives on promoting mental health. In another study, Ackermann and Merrill [55] interviewed 21 participants.

The data for this study were collected through a multiple-method qualitative approach comprised of semi-structured interviews with agricultural specialists and focus group discussions with farmers. The selection of agricultural specialists started with purposeful sampling and then progressed to theoretical sampling [56]. Theoretical saturation was reached after 12 interviews with agricultural specialists in August 2019. Therefore, enrolment of participants, including additional agricultural specialists, was discontinued, as we did not observe any new data or information. Given that the farmers are the main users expected to adopt WSN technologies, four focus group discussions were held with informant farmers. They were considered field specialists who could reflect on the usefulness of WSNs in the field. A semi-structured questionnaire was prepared to guide the interviews and focus group discussions (Table 2). All interviews were conducted in Persian, the native language of the participants, and the interview sessions lasted between 60 and 120 minutes.

**Table 2:** Semi-structured interview questions.

Interview questions
1. How do you know WSNs?
2. Do you see Khuzestan as a potential area for adopting WSNs? Why or why not?
2. What are the factors affecting Khuzestan in adopting WSNs?
3. Do you think that the infrastructure and facilities in Khuzestan are adequate to adopt WSNs?
4. How can the government support WSN adoption in terms of promoting it within Khuzestan?
5. What are the human resource issues related to WSNs adoption in Khuzestan?
6. Do you think that the farmers should be specifically trained to adopt WSNs?
7. How does the economic situation of farmers in Khuzestan affect WSN adoption?
8. How can the private and public sectors be encouraged to support the promotion of or investment in WSNs?
10. How can agricultural service centres/cooperatives provide farmers with WSNs more effectively?
11. Can you think of any other factors that could be a barrier to the adoption of WSNs?

Ethical clearance was first obtained from the review board of the University of Tehran (UT). Following endorsement by the UT, the Jihad Agricultural Organization (JAO) of Khuzestan was informed about the objectives of the study through a support letter from UT. After reviewing the proposal, the JAO wrote some permissions and support letters to the Jihad Agricultural Department in Ahvaz, the Organization of Rural Cooperation in Ahvaz and the Centre for Research and Education in Agriculture and Natural Resources in Khuzestan. Since the current study was non-invasive and used only interviewing of participants, the UT considered that a verbal consent would be adequate. Accordingly, the agricultural specialists and farmers were asked for a verbal consent to confirm their informed voluntary participation. The ethical issues of this study were reviewed and approved based on the research law in Iran. Research participants take part voluntarily, free from any coercion. The confidentiality of the information provided by the survey participants and the anonymity of the respondents were respected. Research participants were fully aware about the purpose and method of the research. The research tool was designed based on the research objectives with complete accuracy and the data were collected by the graduates in complete accuracy.

All interviews were transcribed verbatim. First, content analysis, which is widely used in social science and management research [57], was carried out on the notes taken during the interviews to assess the status of WSN outreach. Next, a three-step analytic process was used to analyse the collected data and identify the barriers to WSN outreach. In the first step, based on the results of a content analysis, the concepts were classified into themes and categories of the different barriers to WSN adoption. Open coding was used to identify variations within the categories and to combine closely related categories in which overlap was found. Following the open coding process, we compared and refined the findings and developed subcategories. In the next step, axial coding was done which means that after merging, removing and reducing the number of categories as needed, the relationships between them were studied. In the third and final step, by selective coding via saturation of the categories and subcategories, a core category was identified, and then other categories were linked to it [58]. After revisiting the categorised statements and identifying the relationships between them, a framework was created. The results are shown in Table 4 and Fig 4.

Both semi-structured interviews and focus group discussions were intentionally selected by researchers for the purpose of data triangulation. Morse [59] suggested that mixing individual interviews and focus groups in a single study increase the validity of study findings through triangulation. In this study, data gathered from semi-structured interviews and focus group discussions were triangulated with direct observation, field notes and prolonged engagement in the subject matter. This approach follows the one presented by Mohajan [60]. It was important to check if the answers to the research questions different depending on the information source. Given that the information is given by different people on the same research questions, enriches our analysis. Hence, the findings of the three data sources (interviews, focus group discussions and field observations) were checked to either align, supplement or contradict.

To allow for the replication of this study, the data coding was added as a S1 File and the interview questions are included in section “2.1 Study design”. Regarding the raw data file of focus group discussion and interviews, unfortunately, the recorded voices and notes taken during the interviews cannot be made publicly available as they contain sensitive and confidential data, the specific names of the organizations and/or political persons, which may lead to further contradictions or ethical issues. Further, the verbal consent for the study stated that “only the study team members will have access to the transcripts." Thus, we are not permitted to share recordings and transcripts of the focus group discussions and interviews.

### 3.2 Study site

This study was conducted in the Khuzestan province that is located in the southwest of Iran. The region covers an area of 64,236 km^2^ and comprises 3,740 villages. The province is a major producer of strategic foods (cereals) and export crops (sugar cane) in the country (representing 33% of the total Iranian cereal production and 52.1% of the total Iranian export crop production) [61]. The adoption of digital and improved agricultural technologies is considered relevant in the Khuzestan context as the province has been a pioneer in the use and extension of new technologies like mechanisation technology in the past [62]. However, WSNs have not yet been used in the province.

## 4 Results and discussion

### 4.1 Interviewees and themes

Table 3 summarises the main characteristics of the interviewed specialists and farmers. Respondents were between 20 and 80 years old indicative of a mix of experience. Out of the 33 respondents, 31 were male and respondents had on average 20 years of work experience. Regarding educational attainment, most of the agricultural specialists had a master’s degree and most of the farmers had a high school diploma. The farmers were therefore considered able to apply and understand the use of WSNs. The agricultural specialists included agricultural experts, agricultural academics and administrators. Wheat, vegetables and rice were the main crops cultivated by the farmers.

**Table 3:** The characteristics of the agricultural specialists and farmers who participated in the interviews and focus groups.

Participants	Specialists (n = 12)												Farmers (Focus group = 4)																				
Features	no. 1	no. 2	no. 3	no. 4	no. 5	no. 6	no. 7	no. 8	no. 9	no. 10	no. 11	no. 12	Group 1 (n = 8)								Group 2 (n = 5)					Group 3 (n = 4)				Group 4 (n = 4)			
													no.1	no.2	no.3	no.4	no.5	no.6	no.7	no.8	no.1	no.2	no.3	no.4	no.5	no.1	no.2	no.3	no.4	no.1	no.2	no.3	no.4
Age (mean)/year	32	48	54	52	53	28	50	32	53	49	38	35	68	50	38	80	50	52	58	35	32	20	60	43	38	27	32	45	58	35	30	36	43
Sex (1: Male; 2: Female)	1	1	1	1	1	2	1	2	1	1	1	1	1	1	1	1	1	1	1	1	1	1	1	1	1	1	1	1	1	1	1	1	1
Job experience (mean)/year	12	20	30	26	30	2	25	12	28	29	13	7	30	5	15	60	30	25	30	13	15	5	30	25	20	7	15	20	27	12	10	8	25
Education																																	
Illiterate																							x										
Elementary																	x	x			x	x									x		
High school														x	x	x			x	x				x		x			x	x			
Bachelor									x				x												x		x	x				x	
Master’s	x		x	x		x	x	x		x																							
Doctoral		x			x						x	x																					
Main crops	-	-	-	-					-	-	-	-	1	1	1	1	1	1	1	1	3	3	3	3	3	1	3	1	1	2	2	3	2
1: Wheat																																	
2: Vegetables																																	
3: Rice																																	
Major	5	2	4	1	4	1	5	5	1	1	3	2	-	-	-	-	-	-	-	-	-	-	-	-	-	-	-	-	-	-	-	-	-
1: Agronomy																																	
2: Irrigation																																	
3: Agricultural mechanisation																																	
4: Agricultural extension and education																																	
5: Plant protection																																	
Level of management:	1	2	3	1	3	1	3	1	1	1	2	2	-	-	-	-	-	-	-	-	-	-	-	-	-	-	-	-	-	-	-	-	-
1: Expert																																	
2: Academic																																	
3: Administrator																																	

### 4.2 Status of WSN outreach

To assess the status of WSN outreach first, the perceptions of agricultural specialists and farmers on the outreach of WSNs were identified, then the extension process with regard to WSN outreach was explored.

#### 4.2.1 Unfamiliarity with WSNs

**Fig 1:**
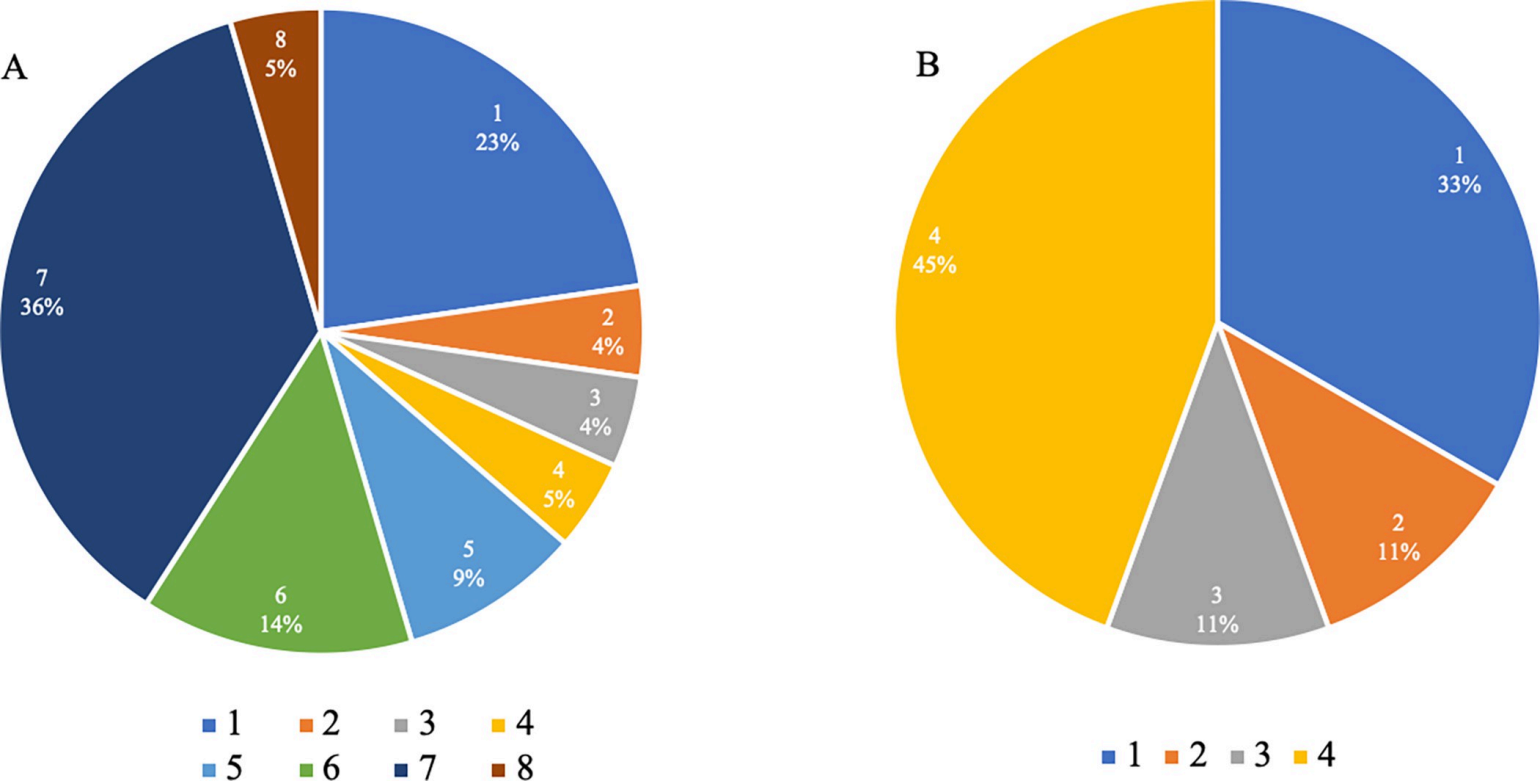
The frequency of the items regarding the outreach of WSNs presented in the interviews (A) and focus group discussions (B) (for the correspondence between the numbers assigned to the items see Table 4).

Table 4 summarises the perceptions of agricultural specialists and farmers we captured on the outreach of WSNs. The frequency of the items presented by specialists and farmers is depicted in Fig 1. According to the figure, the majority of interviewees (7 agricultural specialists) believed that they did not have enough information about WSNs and in all the four focus group discussions, farmers cited no WSNs being used at the farm level in their area. Results of interviews and focus group discussions confirm that both agricultural specialists and farmers were unfamiliar with this technology. In this regard, based on the field notes, interviewee no. 2 stated that:

“*People in rural areas are not familiar with new technologies*. *They are uneducated and traditional and their farming has few innovations*. *Besides*, *WSN is not known to agricultural experts and they are not fully aware of WSN technology*.*”*

**Table 4:** The outreach of WSNs as perceived by agricultural specialists and farmers.

Items	Semi-structured interviews with agricultural specialists (n = 12)	Items	Focus group discussions with farmers (Group = 4)
1	*“I do not have enought information about WSNs*.*”*	1	*“I have no information about WSNs*.*”*
2	*“WSN is an agricultural technology innovation*.*”*	2	*“WSN is the same as agricultural apps on smartphones*.*”*
3	*“WSN is the same as smart farming*.*”*	3	*“WSN seems to be a complex system to learn and implement*.*”*
4	*“WSNs need an internet connection*.*”*	4	*“No WSN is used at the farm level in our area*.*”*
5	*“WSNs display a foundation shift from conventional agriculture*.*”*		
6	*“WSN is considered for large-scale farms*.*”*		
7	*“No WSN is used at the farm level in Khuzestan*.*”*		
8	*“We will think about the WSN in the next 20 years*.*”*		

Despite the unfamiliarity of both agricultural specialists and farmers with WSNs, according to the researchers’ engagement in the subject, Iranian researchers have done numerous studies on this issue and several yield monitoring systems have been developed for crops such as sugar beet [63], sugar cane [64] and potatoes [65]. In the study of Adab et al. [66], remote sensing data was used to retrieve surface soil moisture content in the semi-arid region of Iran. The dataset derived from satellite sensors combined with auxiliary spatial data provided valuable soil moisture content estimation. It showed promise in precision agriculture application. However, the application of WSNs is mostly limited to these academic studies and limited practical WSN experiences are found.

4.2.2 Who has to disseminate WSNs?

The results of the interviews and focus group discussions reveal that farmers have not yet adopted WSNs. They are not familiar with the potential use of WSNs nor are they searching for innovations themselves. The respondents attribute this unfamiliarity to several actors in the extension chain. Interviewees differ in opinion on who should be responsible to gather and transfer information on new technologies. Illustrative are the following quotes.

Focus group discussant no. 4 asserted that:

“*The WSN must be distributed by the government*. *Farmers expect the Agricultural Extension and Service Centre to provide the farmers with appropriate knowledge of WSNs and facilitate farmers’ decisions whether or not and how to adopt WSNs to achieve the best results*.*”*

Yet, while farmers hope to receive the information from the Agricultural Extension and Service Centre, they, in turn, move this responsibility to a higher agricultural department as illustrated by the following quote by the interviewee no. 9 who was working at Agricultural Extension and Service Center:

“*The Agricultural Extension and Service Centre is not responsible to bring the new agricultural technology innovation*, *like WSNs*. *It only shares the information*, *knowledge and farming input obtained from the Jihad Agricultural Department in Ahvaz*.*”*

Yet, the agricultural department also moves the responsibility to the next level; interviewee no. 4, who was an agricultural expert at an agricultural department, said:

“*In regard to new agricultural technologies*, *the Jihad Agricultural Department in Ahvaz follows the Jihad Agricultural Organisation of Khuzestan which is connected to the research centres and is an intermediary between researchers and farmers*.*”*

Interviewee no. 8, who was an agricultural expert at the agricultural organisation, stated that:

“*Because WSNs have only recently come to the attention of researchers*, *few pieces of research are available*. *The centre for Research and Education in Agriculture and Natural Resources in Khuzestan is responsible for trialling and extending WSN systems at the field level*.*”*

And in response to the above challenge, interviewee no. 2, who was a researcher, claimed:

“*Manufacturing*, *launching*, *and applying WSNs needs funding by the government at the national level (Agricultural Research*, *Education and Extension Organisation and Ministry of Agriculture Jihad)*. *The WSN sites are developed in pilot farms in the country in search of more participatory approaches for the dissemination of WSNs*.”

**Fig 2:**
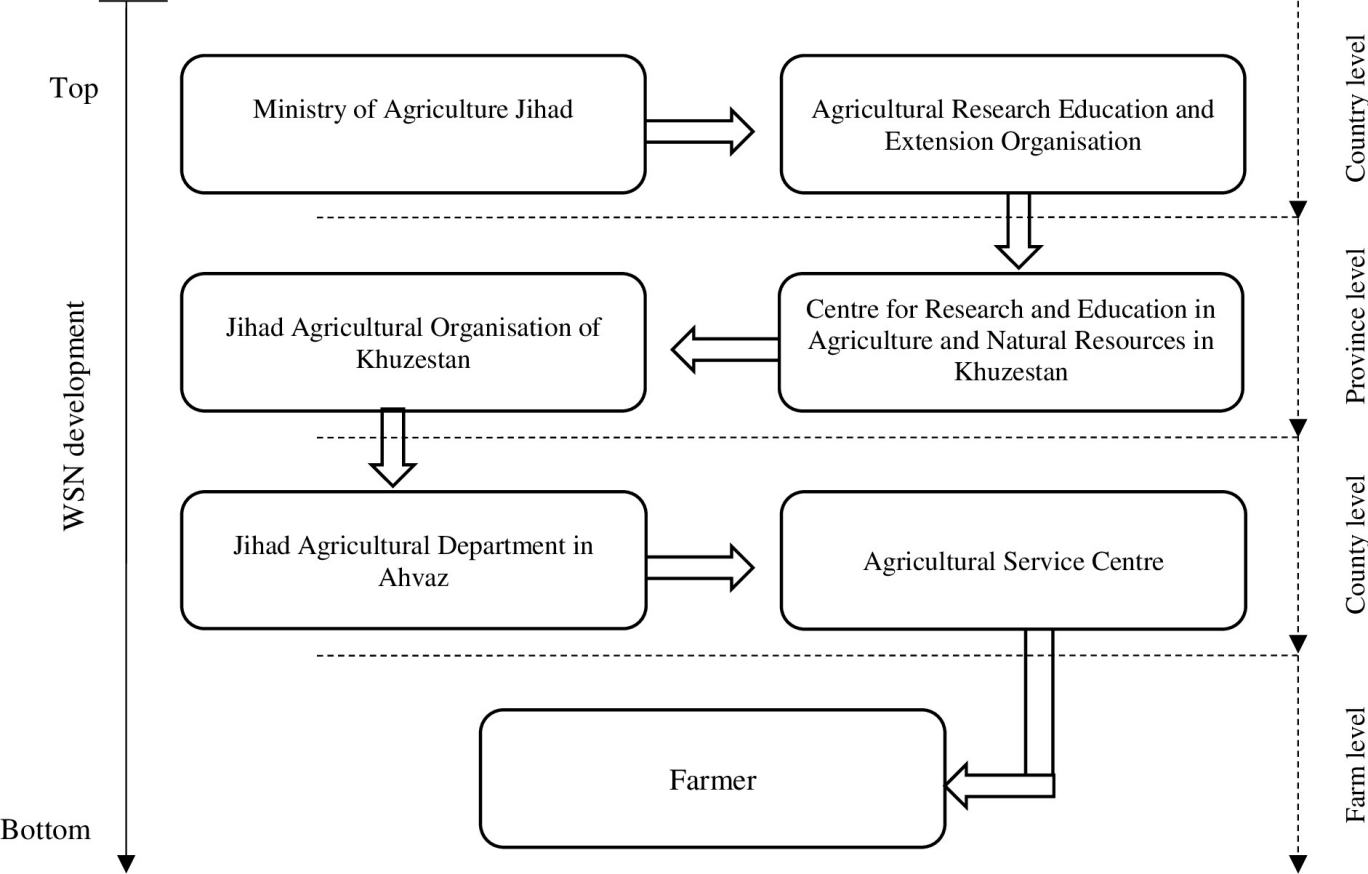
The expected process of WSN outreach from top to bottom (source: field notes and field observations).

In line with the results of interviews and focus group discussions, field observations and the field notes confirm that WSNs are expected to follow a top-down development and extension process in which farmers rely on information from the agricultural extension services, which within their organisation await action from higher-level authorities (Fig 2). The provision of agricultural extension services in Iran is highly centralised and in principle considered the responsibility of the state. In such a centralised network, one or a few powerful actors are dominant nodes and dictate, or at least filter the extension programmes and relevant executive procedures based on their own way of thinking, goals, priorities and available resources [67]. For example, as the most central node in the studied network, the Ministry of Agriculture Jihad decides to a large extent what kind of agricultural technology should be acquired and disseminated in the farming societies. Consequently, farmers, some other governmental agencies and even private institutions have been referring multiple times to the Ministry of Agriculture Jihad. This organisation is powerful and plays a critical role in enhancing or even restricting the sustainability and efficiency of the technological extension system that is supposed to disseminate information on new technologies. As a result, this centralised network provides little operational flexibility and at times lacks the creativity to launch new strategies towards the extension services of new technologies. That might be the reason why the respondents moved the responsibility of not knowing WSNs to the next level in the extension chain.

### 4.3 Barriers to WSN outreach

Based on the content analysis of what farmers and agricultural specialists mentioned during their interviews and focus group discussions, the different barriers to WSN outreach were identified and categorised. Barriers mentioned were attributed to the technology itself, the farmers and the government (Table 5). All the respondents cited a lack of information and awareness among farmers as a major obstacle to the outreach of WSNs, ranking it first, followed by concerns about the high initial cost of WSNs. The lack of encouragement and subsidies by the government was ranked third. The difficulty of learning and implementing, the lack of WSN availability and the lack of awareness and absence of training by extension agents were other important factors mentioned.

**Table 5:** Results of the content analysis of interviews with specialists and focus group discussions with farmers.

Categories	Subcategories	Barriers mentioned	Frequency (%)		Average (%)	Rank
			Focus groups (4 groups)	Interviews (n = 12)		
Technology-related barriers	Complexity of WSN usage	1. Demanding competence in the use of hardware	50.00	16.66	33.33	9
		2. Demanding competence in the use of software	50.00	25.00	37.5	8
		3. Demanding great expertise and technical knowledge	100.00	33.33	66.66	5
		4. Difficulty of learning and implement	75.00	50.00	62.50	4
		5. Having limitation in usability and functional benefits	25.00	16.66	20.83	10
		6. Not appropriate for all farm contexts and sizes	75.00	33.33	54.16	6
	Cost of WSNs	7. High initial costs	100.00	58.33	79.16	2
		8. High operational costs	25.00	33.33	29.16	8
		9. High maintenance costs	25.00	25.00	25.00	9
		10. Not worth to invest	-	16.66	16.66	11
		11. Unclear added value	100.00	33.33	66.66	5
	Lack of availability and accessibility	12. Lack of availability	100.00	41.66	70.83	4
		13. Lack of required equipment (sensors nodes)	100.00	25.00	62.50	6
		14. Not having mobile phone by every farmer	-	16.66	16.66	11
	Reliability of WSNs	15. Low reliability of data	75.00	8.33	41.66	9
		16. Low data security	-	8.33	8.33	12
		17. Possible system errors	50.00	33.33	41.66	7
		18. Not interoperable and not precise enough	75.00	16.66	45.83	8
	Lack of communication-information	19. Lack of internet connection at the farms	50.00	33.33	41.66	7
		20. No internet network coverage at the location of farmers’ house	-	16.66	16.66	11
		21. Low speed available for communication technologies	50.00	33.33	41.66	7
Farmer-related barriers	Rigidity to change	22. Traditional farming practices	25.00	50.00	37.50	6
		23. Resistance and rigidity of farmers	-	16.66	16.66	11
		24. Risk-adverse farmers	25.00	25.00	25.00	9
		25. Low literacy of farmers	50.00	33.33	41.66	7
	Lack of knowledge and skill	26. Lack of information and awareness among farmers	100.00	66.66	83.33	1
		27. Lack of knowledge among leading farmers	50.00	-	50.00	11
		28. Not having enough technical knowledge	75.00	41.66	58.33	5
		29. Difficult to apply WSNs without expert assistance	50.00	50.00	50.00	5
		30. No ability to integrate data collected by WSNs	-	33.33	33.33	9
Government-related barriers	Lack of governmental support	31. No encouragement or subsidy by the government	100.00	50.00	75.00	3
		32. Lack of government financial support (credit and loan)	100.00	41.66	70.83	5
		33. No investments by the government	-	50.00	50.00	7
	Lack of extension and training programmes	34. Lack of local technical expertise and assistance	75.00	41.66	58.33	5
		35. Lack of awareness and absence of training by extension agent	100.00	41.66	70.83	4
		36. No extension programmes regarding WSNs	25.00	25.00	25.00	9
		37. No link between WSNs provider and small-scale farmers by extension agent	100.00	16.66	58.33	7
		38. No support from the agriculture extension establishments and initiative	100.00	25.00	62.50	6
		39. No WSN demonstration plots	100.00	16.66	58.33	7

**Fig 3:**
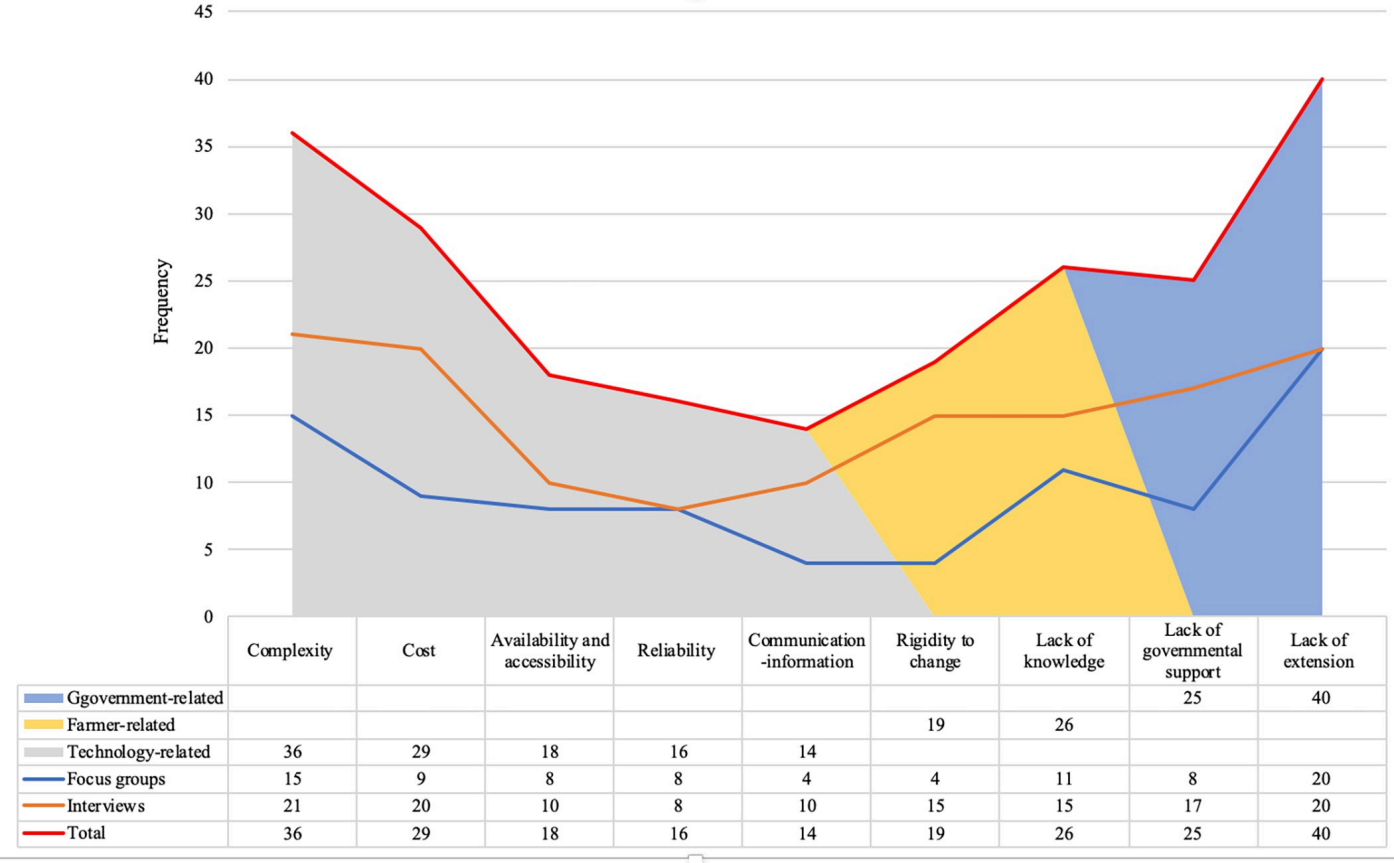
The number of barriers to WSN outreach mentioned by the respondents.

The frequency of the identified barriers to WSN outreach is illustrated in Fig 3. Based on both interviews and focus group discussions, lack of extension and training programmes, complexity of WSN usage and lack of knowledge were the most mentioned statements.

#### 4.3.1 Technology-related barriers

Farmers and specialists mention various technology-related barriers, explaining the current limited use of WSNs. A first concern is the knowledge intensity of WSN applications and the belief that it is a complex system to learn and implement. Interviewees equally referred to the high cost and market availability of WSNs as a barrier to WSN application. High initial costs were mentioned by farmers and specialists. Specialists also expressed concerns about the operational and maintenance costs and that WSNs might be quite expensive for farmers, or as expressed by interviewee no. 6:

“*WSNs need more equipment*, *which may increase the cost of production*.”

This includes both the investment as well as operational costs as expressed by interviewee no. 7:

“*In terms of software and hardware*, *WSNs components are not available in domestic markets and they are also very costly*.*”*

In addition to knowledge, investment and operational costs, respondents were also concerned about the reliability and transfer of data collected by WSNs. Some interviewees believed that the data from WSNs would not be precise enough. While farmer representatives did not refer to the data security of WSNs, one of the agricultural specialists expressed concerns about security and privacy issues, as expressed by interviewee no. 4:

“*The security of WSN data is one of the substantial issues for the farmers*, *i*.*e*., *data suppliers and also end users of WSNs*, *who have low trust in the usage of the data by WSN providers*. *Providers of WSNs must guarantee data security and privacy*.*”*

Inappropriate communication-information infrastructures were also mentioned, and they pointed to issues such as lack of internet connection on the farms, the location of farmer houses and low-speed connections in the area. The interviewees also believed that the profitability of WSNs for farmers was not proven, which would explain why WSNs are not being practised yet. On this point, the discussant of focus group no. 1 stated that:

“*If WSNs meet the perceived needs of farmers and there are enough incentives to encourage their outreach*, *they can quickly take up*.*”*

#### 4.3.2 Farmer-related barriers

The majority of interviewees stated that farmers lack information and are unaware of the possibilities to use WSNs. As an illustration, farmers in the second focus group agreed that:

*“Farmers lack information on farming practices*, *production and recognising different WSN markets and the channels of distribution*.”

Moreover, and fundamentally, the interviewees argued that WSNs involve changing the attitude of all stakeholders about conventional farming practices. Such shifts in attitude may be very difficult to carry through. Farmers explained that digital innovations are not common in the agricultural community. Most of the farmers in rural areas are engaged in traditional farming in which they barely use modern technologies. It is interesting to observe from Table 4 that specialists are more critical of the barriers at the farmers’ level compared to farmers themselves. The specialists mentioned that farmers may be reluctant to change while farmers themselves do not see this as a problem.

#### 4.3.3 Government-related barriers

The respondents pointed to several supportive barriers. Regarding governmental support, low government investment was mentioned as the main obstacle for WSN outreach. The seventh interviewee said:

“*The government has cut most subsidies for agriculture as a result of targeted subsidy policy*. *Accordingly*, *the cost of production has dramatically increased*, *which most framers cannot afford*. *Moreover*, *in terms of investment in this market*, *private enterprises are hesitating due to low productivity in agriculture*. *Therefore*, *to adopt WSN*, *the agriculture of Iran requires a high level of government incentives and support*.*”*

While the agricultural specialists interviewed argue that financial support should focus on providing WSN equipment, facilitation of access to credit and financing options and creation of insurance services, farmer representatives believe that direct payments and subsidies are crucial elements. Focus group discussant no. 4 reported along this line:

“*The government remains responsible for changing farmer attitudes towards WSNs and motivating them to use WSNs*. *A WSN is expensive and small farmers do not have enough money to buy this equipment*. *Providing direct payments and subsidies to farmers to buy WSN equipment and inputs is a necessary factor for the adoption of WSN*.*”*

Most of the interviewees believed that motivated and continuous extension and education services are prerequisites to promote WSN outreach. The limited outreach of WSNs is attributed to the absence of such services. Focus group discussant no. 3 reported in this line:

“*When deciding to adopt and implement WSN principles*, *farmers need to observe the performance of WSNs in the farm situation*. *WSN demonstration plots can provide an excellent opportunity for farmers to observe the performance of WSNs*.”

The interviewees, therefore, believe that extension services are needed to increase the outreach of WSN technology. Among other government extension organisations, agricultural cooperatives have been identified as promising actors in promoting the outreach of WSN technologies as they may help to access the technology and information. The interviewee no. 3 made this argument as follows:

“*Farmer membership in cooperatives enhances their probability of implementing WSNs because they have greater access to resources like credit and extension*. *In addition, when a cooperative adopts WSNs*, *it attempts to generate knowledge that enables production and marketing planning, communication, and monitoring, to keep the cost of WSNs at a low level*.”

## 5 In sum, facts and fears

**Fig 4:**
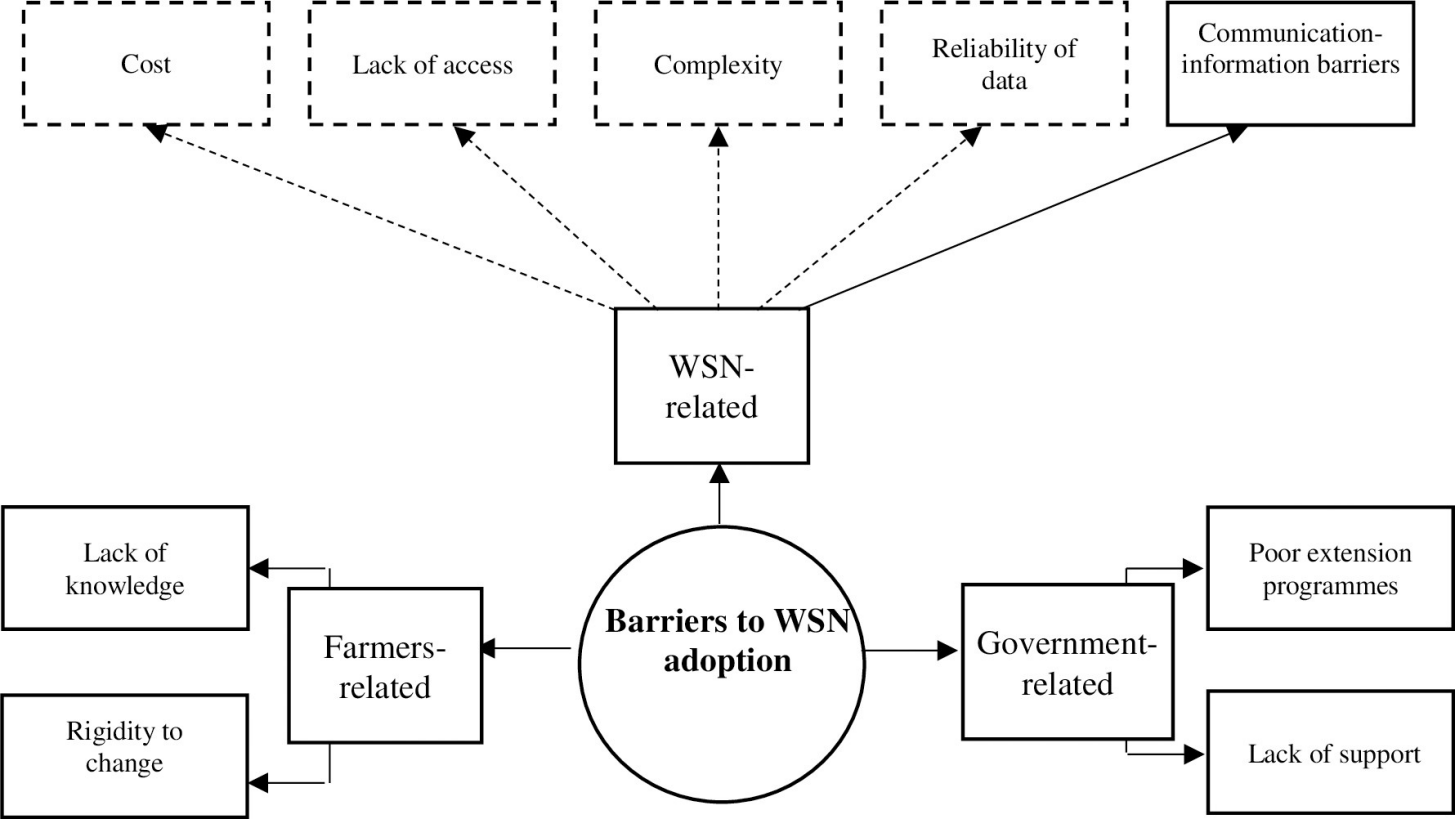
Summary of barriers to WSN outreach as facts and fears (the solid arrows represent the facts, and the dashed arrows represent the fears of adopting WSNs).

Fig 4 summarises the three groups of barriers of WSN outreach discussed above, namely government-related, farmer-related and WSN-related barriers. Yet, what is really crucial is how the respondents understand and perceive such barriers. How respondents explain barriers to WSN outreach does not just show the facts and rational arguments; it also shows the fears and perceptions of risks. The solid arrows in Fig 4 represent what we assume to be facts and inappropriate contextual conditions for WSN outreach. The dashed arrows represent the fears and anxieties of using WSN.

### 5.1 Fears: Technology anxiety

Technology anxiety is defined as an individual’s apprehension and fear when he/she is faced with the possibility of using a technology [68]. Anxiety is generally not caused by a reasonable risk analysis, rather by the unreasonable belief that the technology might turn against the users [69]. We find the following types of fears, namely WSN complexity, WSN reliability, WSN availability and fear of WSN cost.

#### 5.1.1 WSN complexity

A general belief among the respondents was that a WSN is a complex system to learn and implement. This result is in contrast with the findings of several studies [10–13, 70, 71] that showed WSN technology is already being used by many farmers in other countries and a WSN is a well-defined and easy-to-use way to collect data from the field. Accordingly, perceived WSN complexity was considered as one of the obstacles to WSN outreach. This result is in line with the findings of Rezaei-Moghaddam et al. [72], who found that the ease of use positively affects attitudes towards using precision agriculture technologies.

#### 5.1.2 WSN reliability

While WSNs have not yet been implemented at the farm level, and the respondents were quasi unfamiliar with this technology, they surprisingly believed that the reliability of data collected by WSNs is low and that the data would not be precise enough. Hartl and Li [73] calculated that the mean absolute error is 6.17% in traditional WSNs. However, there are different approaches to the design of WSN protocols to enhance the reliability of data transmission [74]. In view of this, several reliability protocols have been proposed in the existing literature [75, 76].

#### 5.1.3 WSN availability

The respondents claimed that WSN components were not available in the market. The main requirement for WSN infrastructure is the availability of the sensor nodes as well as the effective availability of the network for a given application [77]. Several sensor node suppliers were found in the research area. The technology was available online as well.

#### 5.1.4 WSN cost

The respondents perceived WSNs as a high-cost technology. This contradicts the findings of a study conducted by Shinghal and Srivastava [9], who consider a WSN to be low-cost and cost-effective technology. Sanchez-Matamoros et al. [71] also describe WSNs as low-cost and low-consumption technology.

In line with our results, a significant body of previous research has highlighted anxiety as one of the barriers to the adoption or use of new technology [78]. These studies indicate that people with higher levels of technology anxiety intend to use new technologies less. A vital tool to overcome the fears of and to elicit pleasure from novelty instead is collective learning. Palis [74] investigated the role of collective learning in the outreach of integrated pest management methods among farmers in the Philippines. He found that the technical knowledge acquired as a group through experiential learning enabled farmers to overcome their fears, especially those associated with economic risks.

### 5.2 Facts: Inappropriate contextual conditions

Contextual conditions are the specific conditions in which processes, interactions and strategies of action/reaction are taking place; in fact, the circumstances in which a phenomenon occurs [79]. In this research, the contextual conditions are formed by a few subcategories that include poor communication and information, lack of knowledge and skills, farmers’ rigidity to change, lack of governmental support and lack of extension and training programmes.

#### 5.2.1 Poor communication-information

Inappropriate communication and information infrastructure was a barrier that the interviewees referred to. In this regard, they pointed to the low-speed internet connection on the farms and at the location of farmers’ houses. Iran is among the countries with the highest speed of telecommunication development (more than 20% growth rate). The country has also extended telecommunication services to rural areas [80]. In rural areas of Iran, telecommunication technologies have been adopted as a viable alternative to address the inequality in various contexts like access to health services [81]. However, despite the growing global research and development investments in the field of agricultural monitoring, telecommunication technologies for agricultural technology development have not yet received enough attention.

#### 5.2.2 Lack of knowledge and skill

The lack of awareness and absence of knowledge of WSNs among farming communities was another major obstacle to its outreach. According to Alibaygi et al. [82], the lack of relevant and sufficient agricultural information by small-scale farmers is one of the main factors constraining efforts to improve agriculture in Iran. Similarly, Bagherpour and Mohamadi [83] identified a lack of farmers’ knowledge about precision farming as the main challenge for applying this technology in Iran. Several studies reported that increased training and technical support are necessary for improving farmers’ knowledge and the development of applicable technology and facilitating outreach among farmers [84, 85]. Increasing information dissemination and awareness among all WSN stakeholders, especially farmers and policymakers, on the WSN’s benefits is, therefore, an important factor.

#### 5.2.3 Farmers’ rigidity to change

According to the respondents’ perception, farming in the research area is dominated by traditional farming practices. Resistance and rigidity were, therefore, two major hurdles to the outreach of precision farming. In this respect, Bagherpour and Mohamadi [83] and Soltani et al. [86] pointed out that despite the advent of modern agricultural technologies, most Iranian farmers are engaged in conventional and traditional methods for activities like sowing, irrigation, weeding and cultivation with limited use of modern technologies [86].

#### 5.2.4 Lack of government support

The respondents believed that the government does not support WSN outreach. Lack of credit is considered one of the most important challenges of WSN outreach. According to Ardekani et al. [87], as a result of targeted subsidies reform in Iran, the government has cut most of the incentives to agriculture. Similarly, Simtowe and Zeller [88] stated that access to credit facilitates the outreach of risky technologies through relaxation of the liquidity constraints as well as through the boosting of households’ risk-bearing ability. Vaiene et al. [89] have also reported that financial support to farmers can help to stimulate the outreach of agricultural technology.

#### 5.2.5 Lack of extension and training programmes

The limited availability of local extension and training services and assistance was another obstacle for WSN outreach. The findings of a study by Alimirzaei et al. [90] showed that the current executive network in Iran is not satisfactory, such that a few dominant providers are very influential in the centre, while most of the others have little power at the margin of the extension network. According to Mwangi and Kariuki [91], availability and access to extension services have been found to be key aspects of the outreach of technology. Many authors have reported a positive relationship between extension services and technology outreach [91, 92]. Important is that extension activities should be able to provide farmers with up-to-date information through farmer exchange visits and informal farmer-to-farmer interactions. In this regard, the public sector has a key role to play here in ensuring that the extension services deliver high-value information to farmers.

## 6 Conclusion

This study gives insights challenges of WSN outreach. Understanding these challenges can be a basis for developing WSNs policy interventions to encourage the outreach of WSNs by farmers to encourage the outreach of WSNs by farmers in Iran and other developing countries. The results revealed that the use of WSNs by farmeers is still in an early stage. The application of WSNs is mostly limited to academic studies and WSN is not yet practiced at farm level. The findings of the study showed that WSN outreach barriers can be classified into three groups: government-related, farmer-related and WSN-related barriers. Some of the identified barriers show the facts and inappropriate contextual conditions for WSN outreach. Other barriers reflect the fears and perceptions of risks of adopting WSNs.

Low encouragement and the lack of subsidising support measures by the government, a lack of information and awareness among farmers and the cost of WSNs were mentioned as the main obstacles to WSN outreach. The Iranian government has not devoted significant efforts to promoting WSNs. Most of the farmers are small-scale farmers, are often poor and have no knowledge and poor access to WSNs. The high initial cost of WSNs is also a fear factor for its outreach by farmers. Also, the current extension system seems not conducive to WSN outreach, and the respondents move the responsibility of not knowing about WSNs to the next level in the extension chain. Some actors in the extension system seem to dominate the technology choices in the extension service and are very influential, resulting in a highly centralised network. If WSNs shall expand faster in the future, then action is expected from the government of Iran, as it is their key responsibility to invest in an appropriate strategy to overcome the barriers identified in this study. An important structural adjustment in the current extension system could be a multisectoral institutional platform that focuses on enhancing operational flexibility, creativity and the launching of new strategies for technology extension.

Finally, this study is not without limitations which require further research. First, since there are a number of drivers that can influence WSN outreach and in this study, only government-related, farmer-related and WSN-related barriers are identified, further research is required to determine the other barriers of WSN outreach. Second, in this research, the relationships between the various barriers to WSN outreach have been identified only based on Iranian experts and farmers’ opinions. Therefore, the results may be limited by the level of knowledge and experience of farmers and experts in this area. Since the framework developed is not statistically validated, there is a need for future research to test and validate the model. Third, this study revealed that the current extension system seems not to be conducive to WSN outreach. More studies are critical to understanding how governments can create an enabling extension structure for WSNs’ outreach by considering various challenges associated with the current extension system. The findings of our study also highlight a need for further studies in order to identify the different perceptions of policy makers as well as their opinions on the most effective policies. Fourth, the study is exploratory, and a principal limitation is probably the small number of respondents. Because WSN technology in Iran is passing through a research stage and not many farmers have tried to apply WSNs in practice, reaching a large number of respondents who were able to participate in the study proved extremely difficult. Future studies may increase the sample by including a few country case studies at the same development status which would allow us to understand the differences in perceptions towards the application of WSNs. Finally, as this study focuses on the identification of the barriers, a follow up study may consist of a quantitative survey to assess the distribution of the identified barriers.

## Supporting information

S1 File (DOCX)

## List of abbreviations

JAO: Jihad Agricultural Organization
PA: Precision agriculture
UT: University of Tehran
WSNs: Wireless sensor networks
