# Supplementary material for: Facts and fears that limit digital transformation in farming: Exploring barriers to the outreach of wireless sensor networks in Southwest Iran
Source: PLoS One. 2022 Dec 16;17(12):e0279009. doi: 10.1371/journal.pone.0279009 (PMC9757575; doi:10.1371/journal.pone.0279009)
Supplement: S1 File — (DOCX) [file pone.0279009.s001.docx]

**Summary of the categories and data coding**

| Categories Code | Categories | Subcategories | Concepts |
| --- | --- | --- | --- |
| A | Technology-related barriers | A1: Complexity of WSN usage | 1. Demanding competence in the use of hardware |
|  |  |  | 1. Demanding competence in the use of software |
|  |  |  | 1. Demanding great expertise and technical knowledge |
|  |  |  | 1. Difficulty of learning and implement |
|  |  |  | 1. Having limitation in usability and functional benefits |
|  |  |  | 1. Not appropriate for all farm contexts and sizes |
|  |  | A2: Cost of WSNs | 1. High initial costs |
|  |  |  | 1. High operational costs |
|  |  |  | 1. High maintenance costs |
|  |  |  | 1. Not worth to invest |
|  |  |  | 1. Unclear added value |
|  |  | A3: Lack of availability and accessibility | 1. Lack of availability |
|  |  |  | 1. Lack of required equipment (sensors nodes) |
|  |  |  | 1. Not having mobile phone by every farmer |
|  |  | A4: Reliability of WSNs | 1. Low reliability of data |
|  |  |  | 1. Low data security |
|  |  |  | 1. Possible system errors |
|  |  |  | 1. Not interoperable and not precise enough |
|  |  | A5: Lack of communication-information | 1. Lack of internet connection at the farms |
|  |  |  | 1. No internet network coverage at the location of farmers’ house |
|  |  |  | 1. Low speed available for communication technologies |
| B | Farmer-related barriers | B1: Rigidity to change | 1. Traditional farming practices |
|  |  |  | 1. Resistance and rigidity of farmers |
|  |  |  | 1. Risk-adverse farmers |
|  |  |  | 1. Low literacy of farmers |
|  |  | B2: Lack of knowledge and skill | 1. Lack of information and awareness among farmers |
|  |  |  | 1. Lack of knowledge among leading farmers |
|  |  |  | 1. Not having enough technical knowledge |
|  |  |  | 1. Difficult to apply WSNs without expert assistance |
|  |  |  | 1. No ability to integrate data collected by WSNs |
| C | Government-related barriers | C1: Lack of governmental support | 1. No encouragement or subsidy by the government |
|  |  |  | 1. Lack of government financial support (credit and loan) |
|  |  |  | 1. No investments by the government |
|  |  | C2: Lack of extension and training programmes | 1. Lack of local technical expertise and assistance |
|  |  |  | 1. Lack of awareness and absence of training by extension agent |
|  |  |  | 1. No extension programmes regarding WSNs |
|  |  |  | 1. No link between WSNs provider and small-scale farmers by extension agent |
|  |  |  | 1. No support from the agriculture extension establishments and initiative |
|  |  |  | 1. No WSN demonstration plots |
